# More Than a Watchdog: Harnessing State, Civil Society and Academia to Tackle Unhealth Commodity Industries; A Response to Recent Commentaries

**DOI:** 10.34172/ijhpm.9260

**Published:** 2025-07-19

**Authors:** Elizabeth Bennett, Stephanie M. Topp, Alan Rob Moodie

**Affiliations:** ^1^College of Public Health, Medical and Veterinary Sciences, James Cook University, Townsville, QLD, Australia.; ^2^Melbourne School of Population and Global Health, University of Melbourne, Melbourne, VIC, Australia.

## Introduction

 We thank colleagues for their thoughtful commentaries on our scoping review and framework synthesis of national public health surveillance of corporations in key unhealthy commodity industries (UCIs).^1-7^ Our review aimed to consolidate existing frameworks to monitor the influence of UCIs on health to inform future surveillance efforts and reduce the impact of corporate practices that undermine health.^2^

 Commentators widely acknowledge the importance and urgency of monitoring UCIs and the value our review adds advancing this agenda. Four core themes emerged from the commentaries for further discussion: (1) who should own surveillance, (2) the need for structural and systematic change alongside monitoring, (3) expanding scope of monitoring beyond the “harmful trinity,” and (4) the role of data and technology in enhancing surveillance. Here, we reflect on these insights and propose a future direction for advancing effective, accountable and systematic monitoring of UCIs.

## Who Should “Own” Surveillance? Limits of Government-Led Surveillance

 Authors raise legitimate concerns around limitations of government-led surveillance of UCIs, citing the risk of corporate capture, resource constraints, policy incoherence, and limited political will. Indeed, a prevailing view among the commentaries is that civil society, academia, and independent organisations may be better suited to lead monitoring efforts instead of government bodies.^1,3-7^

 While these are valid challenges, they are not unique to the commercial determinants of health. Similar limitations and patterns affect other areas of public health and have not deterred expectations for government accountability.

 We applaud the leadership that civil society, academia and independent organisations have demonstrated in mobilising public pressure on UCIs, promoting transparency, and making the monitoring more effective. Their role is critical and independent oversight must remain central to any surveillance model.

 However, we argue that this status quo, where responsibility of monitoring is largely external to the state, is insufficient. As Delobelle highlights, new whole-of-government approaches, backed by political commitment and coherent policy, are required.^4^ Models that enable governments to support and fund accountability mechanisms and independent surveillance, as suggested by Lacy-Nichols and Delobelle, offer a pragmatic path forward.^4,7^

 Precedents exist in areas like anti-corruption (eg, anti-corruption commissions), financial regulation (eg, securities commissions), and environmental protection (eg, environmental protection agencies), where state-sponsored bodies operate with legal authority, independence, and public reporting mandates. Applying or adapting such models to UCI surveillance could institutionalise transparency, reduce political interference, and demonstrate that corporate practices harmful to health are under formal scrutiny.^8^ These new models should also embed mechanisms for bottom-up approaches to guide the design and priorities of surveillance systems, anchoring top-down models in public accountability and real-world relevance.^3,5^ Importantly, advocating for government-led surveillance is not only about improving data collection and enforcement capacity. If governments begin systematically monitoring all UCIs as vectors of non-communicable diseases, similar to tobacco, this signals a paradigmatic shift in how their role in public harm is understood. This reframing positions UCIs as systemic risks to health, rather than legitimate stakeholders. Predictably, such moves will face resistance, making political will and institutional autonomy essential, as the commentators highlight. Nonetheless, taking surveillance seriously is a strategic intervention that sends a powerful signal that these industries warrant the same scrutiny as other public health threats.^9,10^

 Incremental approaches, as suggested by Lacy-Nichols and colleagues, may be a pragmatic starting point in politically constrained environments. But ultimately, integrated governance model is needed to match the scale and ubiquity of UCI influence.^7^

## Surveillance Must Be Embedded in Structural and Systemic Change

 As several authors note, surveillance alone is insufficient to reduce corporate harms to health.^1,5,6^ Monitoring must be accompanied by meaningful change to the political and economic structures that enable UCI influence. Surveillance should function as a lever for broader systemic transformation – not just an observational exercise.

 Government-led models are better positioned to deliver this, given proximity to policy levers, and have the potential to deliver a more coordinated and sustained response. Civil society initiatives, while impactful, often face challenges related to scale, sustainability and fragmentation.

## Expanding Scope of Surveillance: Beyond the “Harmful Trinity”

 While our review focused on tobacco, alcohol and ultra-processed food and beverages, commentators rightly urge expansion to other industries.^1,6,7^ As discussed in our article, sectors such as fossil fuels, gambling, pharmaceuticals and technology, increasingly shape health outcomes and policy environments. Surveillance efforts must evolve to include these industries and adapt tools accordingly.

 Baum and Anaf underscore the need to move beyond individual practices and address broader economic and political norms that legitimise harmful corporate influence.^1,11^ Surveillance should be responsive to these interconnections and monitor the synergistic impact of practices across industries.

## Leveraging Data and Technology for More Effective Surveillance

 Since publication of our review, data science and artificial intelligence (AI) tools have rapidly advanced. Commentators highlight the potential of technologies such as web scraping, natural language processing and large language models to automate of data collection and improve pattern recognition.^7^

 These tools can help identify networks of influence, link health outcomes to specific corporate products, and estimate societal costs of UCIs in real time.^6^ Integration into open-access platforms could democratise surveillance, increase transparency and broaden public access to critical information.

## Conclusion

 We thank our colleagues for reinforcing the importance of UCI surveillance that is independent, well-resourced, and rooted in broader structural reforms.

 Civil society and academia have led this work for too long in the absence of meaningful government engagement. But we need more than a watchdog – surveillance of UCIs is not just about collecting data; it is a political act that signals institutional recognition of harm. Governments must now take ownership. Academic and civil society actors can support this transition by offering expertise, tools, and collaborative models that help embed UCI surveillance as a core public health function. In the face of concentrated corporate power, fragmented efforts are no longer enough. Working together, we can build robust and enduring systems that expose harmful practices, hold UCIs to account, and ultimately, protect public health.

## Acknowledgements

 We particularly wish to thank all authors of commentaries to our original article.

## Ethical issues

 Not applicable.

## Conflicts of interest

 Authors declare that they have no conflicts of interest.

## Disclaimers

 The views expressed in this article are those of the authors.
